# Strengths and Weaknesses of Immunotherapy for Advanced Non-Small-Cell Lung Cancer: A Meta-Analysis of 12 Randomized Controlled Trials

**DOI:** 10.1371/journal.pone.0032695

**Published:** 2012-03-05

**Authors:** Juan Wang, Ze-Hong Zou, Hong-Lin Xia, Jian-Xing He, Nan-Shan Zhong, Ai-Lin Tao

**Affiliations:** Guangzhou Municipal Key Laboratory of Allergy and Clinical Immunology, Allergy Research Branch of the State Key Laboratory of Respiratory Disease, The Second Affiliated Hospital of Guangzhou Medical University, Guangzhou, China; Jiangsu University, China

## Abstract

**Background:**

Lung cancer is one of the leading causes of cancer death worldwide. Non-small-cell lung cancer (NSCLC) accounts for approximately 85% of all lung cancers. Immunotherapy has yielded no consistent benefit to date for those patients. Assessing the objective efficacy and safety of immunotherapy for advanced NSCLC patients will help to instruct the future development of immunotherapeutic drugs.

**Methodology and Principal Findings:**

We performed a meta-analysis of 12 randomized controlled trials including 3134 patients (1570 patients in the immunotherapy group and 1564 patients in the control group) with histologically confirmed stage IIIA, IIIB, or IV NSCLC. The analysis was executed with efficacy end points regarding overall survival (OS), progression-free survival (PFS), complete response (CR), partial response (PR), and total effective rate. Overall unstratified OS, PFS, PR, and total effective rate were significantly improved in advanced NSCLC patients in the immunotherapy group (*P* = 0.0007, 0.0004, 0.002, 0.003, respectively), whereas CR was not improved (*P* = 0.97). Subgroup analysis showed that monoclonal antibody (mAb) immunotherapy significantly improved the PFS, PR, and total effective rate and showed a trend of improving OS of advanced NSCLC patients compared with the control group, with one kind of adverse event being significantly dominant. Compared with the control group, the vaccine subgroup showed no significant difference with regard to serious adverse events, whereas cytokine immunotherapy significantly induced three kinds of serious adverse events.

**Conclusions:**

Immunotherapy works efficiently on advanced NSCLC patients. Of several immunotherapies, mAb therapy may be a potential immunotherapy for advanced NSCLC patients, and become a standard complementary therapeutic approach in the future if the issues concerning toxicity and allergenicity of mAbs have been overcome.

## Introduction

In 2008, lung cancer was the most commonly diagnosed cancer, as well as the leading cause of cancer death in males worldwide. Among females, it was the fourth most commonly diagnosed cancer and the second leading cause of cancer death [1]. Non-small-cell lung cancer (NSCLC) accounts for approximately 85% of all lung cancers [2].

Despite recent advances in surgery, irradiation, and chemotherapy, the prognosis of patients with lung cancer is still poor [3]. About 50% of patients recur after surgery, and less than 25% of patients respond to systemic chemotherapy [4]. For patients with unresectable stage III NSCL, chemotherapy has limited benefits [5], [6]. For advanced NSCLC patients, chemotherapy induces significant safety issues. For example, in one study including 1371 patients, of 58% patients who received chemotherapy, 35% had adverse events (AEs) and more than 12% had serious AEs [7].

Thus, it urgently requires safer and more effective treatments for lung cancer to improve the quality and duration of life. Immunotherapy seems an attractive therapeutic approach for lung cancer due to its theoretical specificity and potential for long-term disease control [8]. At present, the main strategies of immunotherapy for advanced NSCLC include vaccines, cytokines, and monoclonal antibodies (mAbs). Vaccine immunotherapy prompts the immune system to kill cancer cells [9], immunotherapy with cytokines counteracts the immunodeficiency state caused by the tumor, and monoclonal antibodies (mAbs) target specific tumor antigens and induce immune response against cancer [10]. However, immunotherapy trials for lung cancer have yielded no consistent benefit to date in humans because tumor cells can escape the immune attack and develop different resistance mechanisms [9]. Combination of immunotherapy with surgery, chemotherapy, or radiotherapy may be valuable in NSCLC patients; nevertheless the model of multi-modality in NSCLC is still being debated.

Meta-analysis based on data from pooled patient samples provides an avenue for evaluating the efficacy and side effects of immunotherapy for advanced NSCLC patients. In this study, we used a meta-analysis to evaluate the efficacy and safety of the immunotherapies (including chemo-immunotherapy) on advanced NSCLC patients.

## Methods

### Literature Search Strategy

**Figure 1:**
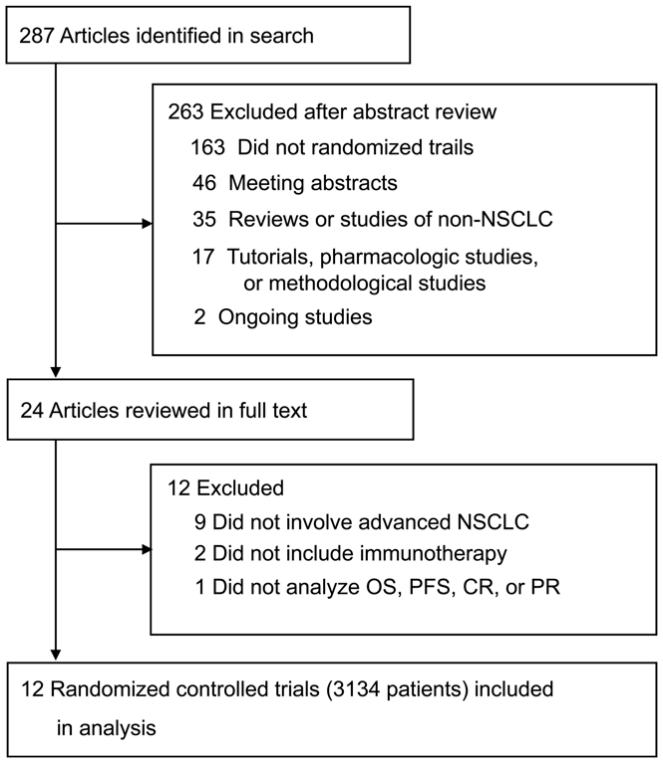
Study Flowchart.

This meta-analysis adhered to the relevant criteria of the PRISMA (Preferred Reporting Items for Systematic Reviews and Meta-Analyses) statement [11]. A search was conducted on Highwire (PubMed included) for original studies published between January 1980 and April 2011 on immunotherapy for NSCLC, using the following keywords: “immunotherapy” OR “immunotherapeutic” AND “non-small-cell lung cancer” OR “NSCLC.” Review papers were also examined for published results. By carefully examining the body of each publication and the names of all authors, we avoided duplications of data. When such duplications were identified, the latest version was included in this study. The search strategy used is illustrated in Figure 1.

### Selection Criteria

The selection criteria were as follows: (1) studies were in the English language and were limited to human trials; (2) data regarding tumors without specific documentation of lung origin were excluded; (3) case studies, review articles, and studies involving fewer than three patients were excluded; and (4) studies adopting randomized controlled trials to compare immunotherapy versus control therapy and including patients at stage IIIA, IIIB, or IV were included.

### Data Extraction and Quality Assessment

Three reviewers, JW, HLX and ALT, independently selected the trials and performed the data extraction. Discrepancies were resolved by discussion among the reviewers. The clinical outcomes used to evaluate efficacy and safety of immunotherapy in advanced NSCLC were overall survival (OS), progression-free survival (PFS), complete response (CR), partial response (PR), and the total effective rate (CR + PR). OS was defined as the period from the randomization date to the date of death. PFS was defined as the period from the randomization date to the date when disease progression (or death) was observed. We assessed the objective cancer response as total effective rate, CR, and PR.

For the meta-analysis of immunotherapy for NSCLC, the overall quality of each study was assessed in accordance with the Jadad Scale [12]. A grading scheme (a, b, and c) was used to classify four main criteria: (1) quality of randomization; (2) quality of allocation concealment; (3) quality of blinding; and (4) quality of the description of withdrawals and dropouts [13]. The grades indicate: (a) adequate, with appropriate procedures; (b) unclear, inappropriate description of methods; and (c) inadequate procedures, methods, or information [14]. Based on these four criteria, each study cited can be categorized as follows: A. studies have a low risk of bias and were scored as grade a for all items; B. studies have a moderate risk of bias with one or more grades of b; and C. studies have a high risk of bias with one or more grades of c.

### Assessment of Safety

For the trials included in this study, different grades of toxicity and serious adverse events (SAEs) were observed during the follow-up periods. An event that was fatal, life-threatening, required hospitalization or prolonged existing hospitalization, or caused a persistent or significant disability/incapacity was defined as an SAE [15]. AEs were graded using the National Cancer Institute Common Toxicity Criteria, version 2.0, except AEs reported by Lissoni et al. [16], which were graded using WHO criteria.

The included trials were classified into three subgroups (cytokines, mAbs, and vaccines) based on the three categories of immunotherapeutic drugs administered for advanced NSCLC. Subgroup analysis of the SAEs was performed using Peto odds ratio [17] to assess the significance of differences between the experimental arm and its control arm in each subgroup.

### Statistical Analysis

Statistical analysis was carried out using Review Manager (version 5.0) provided by The Cochrane Collaboration. Dichotomous data are presented as hazard ratios (HR) and continuous outcomes as weighted mean differences, both with the 95% confidence interval (CI). HR and CI were calculated according to Cox proportional hazards modeling [18]. An HR<1 means a lower rate of events in the maintenance arm [19]. The overall effect was tested using *Z* scores, with significance set at *P*<0.05. Meta-analysis was performed using random-effect or fixed-effect methods, depending on the presence or absence of significant heterogeneity [20]. Statistical heterogeneity between trials was evaluated by the χ^2^ and *I* ^2^ tests, with significance set at *P*<0.10. When heterogeneity was confirmed, the random-effect method was used. In the absence of statistically significant heterogeneity, the fixed-effect method was used to combine the results. Sensitivity analysis was conducted with alternative exclusion of trials by Neninger Vinageras et al. [21] or Butts et al. [22], two trials that did not apply chemotherapy in both the experimental and control arms.

## Results

### Quantity of Evidence

A total of 287 studies were identified by the searches. By scanning titles and abstracts, redundant publications, reviews, meeting abstracts, and case reports were excluded. After referring to full texts, we removed 275 studies that did not meet the selection criteria (Figure 1). As a result, 12 studies [16], [21]–[31] that included a total of 3134 patients were selected for meta-analysis.

The details of the 12 trials are listed in Table 1. Although six studies did not describe OS [23] or PFS [16], [21], [22], [24], [26] and four studies did not provide the number of patients in CR and/or PR rates [22], [26], [29], [31], all 12 studies were open-labeled and randomized. They mentioned the concealment of allocation clearly in the randomization process, and provided the number of patients who withdrew from the trials. Therefore, the 12 studies provided adequate information and were thus considered to be A. studies in this meta-analysis (Table 2).

**Table 1:** Detailed data of the 12 trials included in the meta-analysis.

Source [Reference]	Trial phase	Stage of patients	Follow-up (years)	Study groups	Treatment design	Dosage	No. of patients (*N* = 3134)	No. of events/No. of subjects				
								OS	PFS	CR	PR	TER
Lissoni et al. [16]	Phase II	IIIA/IIIB	3	Exp	MLT + low-dose rIL-2	3×106 IU/day/40 mg/day	29	27	No	0	7	7
				Con	CE	C (20 mg/m2), E (100 mg/m2)	31	29	No	0	6	6
Neninger Vinageras et al. [21]	Phase II	IIIB/IV	4	Exp	EGF vaccinations	50 µg	40	35	No	0	7	7
				Con	BSC	No	40	38	No	2	9	11
Butts et al. [22]	Phase IIB	IIIB/IV	3	Exp	BSC + L-BLP25	1000 µg/No	88	61	No	49	No	49
				Con	BSC	No	83	69	No	45	No	45
Gatzemeier et al. [23]	Phase II	IIIB/IV	1.7	Exp	TGC	T (4 mg/kg)/G (1250 mg/m^2^), C (75 mg/m^2^)	51	No	45	2	16	18
				Con	GC	G (1250 mg/m^2^), C (75 mg/m^2^)	50	No	42	1	20	21
Lasalvia-Prisco et al. [24]	Phase II	IV	1	Exp	GM-CSF + Cyclophosphamide	300 mg/m^2^/300 µg SC	44	21	No	0	14	14
				Con	CHT	D (100 mg/m^2^), C (80 mg/m^2^)	44	29	No	1	15	16
Lynch et al. [25]	Phase III	IIIB/IV	3	Exp	TC + Cetuximab	400 mg/m^2^/T (225 mg/m^2^), C (6, 30-min IV)	338	277	284	0	87	87
				Con	TC	T (225 mg/m^2^), C (6, 30-min IV)	338	287	263	1	57	58
O'Brien et al. [26]	Phase III	IIIA/IIIB/IV	2	Exp	MVP + SRL172	M (8–10 mg/m^2^), V (6 mg/m^2^), C (50–120 mg/m^2^)/0.1 ml	210	197	No	No	No	No
				Con	MVP	M (8–10 mg/m^2^), V (6 mg/m^2^), C (50–120 mg/m^2^)	209	201	No	No	No	No
Pirker et al. [27]	Phase III	IIIB/IV	2.5	Exp	VC + Cetuximab	V (25 mg/m^2^), C (80 mg/m^2^)/400 mg/m^2^	557	468	446	9	194	203
				Con	VC	V (25 mg/m^2^), C (80 mg/m^2^)	568	494	408	6	160	166
Ridolfi et al. [28]	Phase III	IIIB/IV	3	Exp	GC + IL-2	G (1000 mg/m^2^), C (100 mg/m^2^)/3,000,000 IU/die	127	93	111	0	18	18
				Con	GC	G (1000 mg/m^2^), C (100 mg/m^2^)	114	85	99	1	12	13
Rosell et al. [29]	Phase II	IIIB/IV	2	Exp	VC + Cetuximab	V (25 mg/m^2^), C (80 mg/m^2^)/400 mg/m^2^	43	36	28	No	No	15
				Con	VC	V (25 mg/m^2^), C (80 mg/m^2^)	43	40	28	No	No	12
Wu et al. [30]	Phase II	IIIA/IIIB/IV	2.5	Exp	TP + CIK	D (75 mg/m^2^), C (25 mg/m^2^)/1.0×10^9^ cells	29	23	27	0	13	13
				Con	TP	D (75 mg/m^2^), C (25 mg/m^2^)	30	29	29	0	13	13
Zhong et al. [31]	Phase I/II	IIIB/IV	∼5	Exp	NP + peptide-pulsed autologous dendritic cells and CIK cells	V (25 mg/m^2^), C (75 mg/m^2^)/Repeated at 30-day	14	12	13	No	No	No
				Con	NP	V (25 mg/m^2^), C (75 mg/m^2^)	14	13	14	No	No	No

**Note:** 3134 patients were included in the meta-analysis, with 1570 assigned to the experimental groups (Exp) treated with immunotherapy and 1564 in the control groups (Con).

**Abbreviations:** CR, complete response rate; OS, overall survival; PFS, progression-free survival; PR, partial response rate; TER, total effective rate, which is equal to CR plus PR; No, no detailed data; BSC, best supportive care without drug regimen; C, cisplatin; CE: cisplatin + etoposide.; CHT, chemotherapy (docetaxel + cisplatin); CHIMT, CHT + an immunomodulatory adjuvant system; CIK, cytokine-induced killer biotherapy; D, docetaxel; EGF, epidermal growth factor; GC: gemcitabine-cisplatin; GM-CSF, granulocyte macrophage-colony stimulating factor; L-BLP25, BLP25 liposome vaccine; MLT, melatonin; MVP, mitomycin C + vinblastine + cisplatin; NP, vinorelbine-platinum; rIL-2, recombinant interleukin 2; SRL172, killed *Mycobacterium vaccae*; TC, taxane + cisplatin; TCG: trastuzumab + gemcitabine-cisplatin; TP, docetaxel + cisplatin; V, vinorelbine.

**Table 2:** Jadad Scale for the 12 randomized controlled studies.

Author [Reference]	Randomization (grades)	Allocation concealment (grades)	Blinding(grades)	Description of withdrawals (grades)	Category
Lissoni et al. [16]	a	a	a	a	A
Neninger Vinageras et al. [21]	a	a	a	a	A
Butts et al. [22]	a	a	a	a	A
Gatzemeier et al. [23]	a	a	a	a	A
Lasalvia-Prisco et al. [24]	a	a	a	a	A
Lynch et al. [25]	a	a	a	a	A
O'Brien et al. [26]	a	a	a	a	A
Pirker et al. [27]	a	a	a	a	A
Ridolfi et al. [28]	a	a	a	a	A
Rosell et al. [29]	a	a	a	a	A
Wu et al. [30]	a	a	a	a	A
Zhong et al. [31]	a	a	a	a	A

a : adequate, with correct procedures;

b : unclear, without a description of methods; and.

c : inadequate procedures, methods, or information. A studies have a low risk of bias and were scored as grade a for all items.

### Meta-Analysis of Immunotherapy for Advanced NSCLC

**Figure 2:**
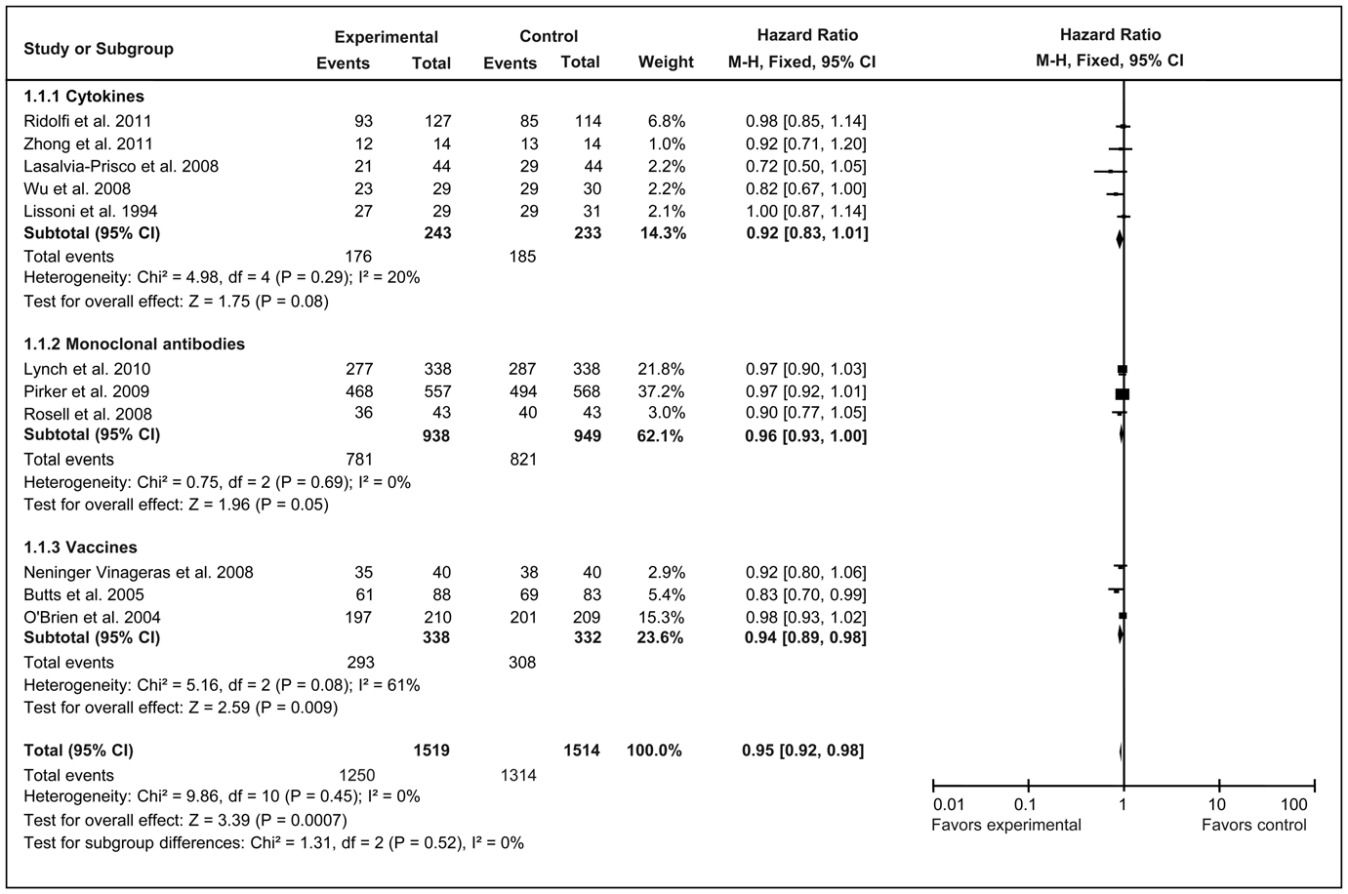
Forest plot of comparison of overall survival of 11 included studies (Stage IIIA, IIIB, or IV NSCLC). *P* values are from P-for-effect modification testing for heterogeneity within or across the groups of regimens. The sizes of data markers are proportional to the number of deaths in the trials. CI, confidence interval; HR, hazard ratio.

The analysis results of OS are shown in Figure 2. No significant heterogeneity was detected for total unstratified immunotherapy or the three subgroups defined by immunotherapeutic categories (Figure 2). A fixed-effect model was therefore used for OS analysis. The overall analysis showed that immunotherapy significantly increased OS at the end of follow-up compared with the control group (*Z* = 3.39, *P* = 0.0007). However, subgroup patients did not consistently gain an OS benefit from the various immunotherapies. The vaccine group behaved the same as total unstratified immunotherapy and improved OS significantly (HR = 0.94, 95% CI = 0.89–0.98; *Z = *2.59, *P* = 0.009), whereas the cytokine group (HR = 0.92; 95% CI = 0.83–1.01; *Z = *1.75, *P* = 0.08) and mAb group (HR = 0.96, 95% CI = 0.93–1.00; *Z = *1.96, *P* = 0.05) did not produce any significant improvement in OS compared with their corresponding control groups.

**Figure 3:**
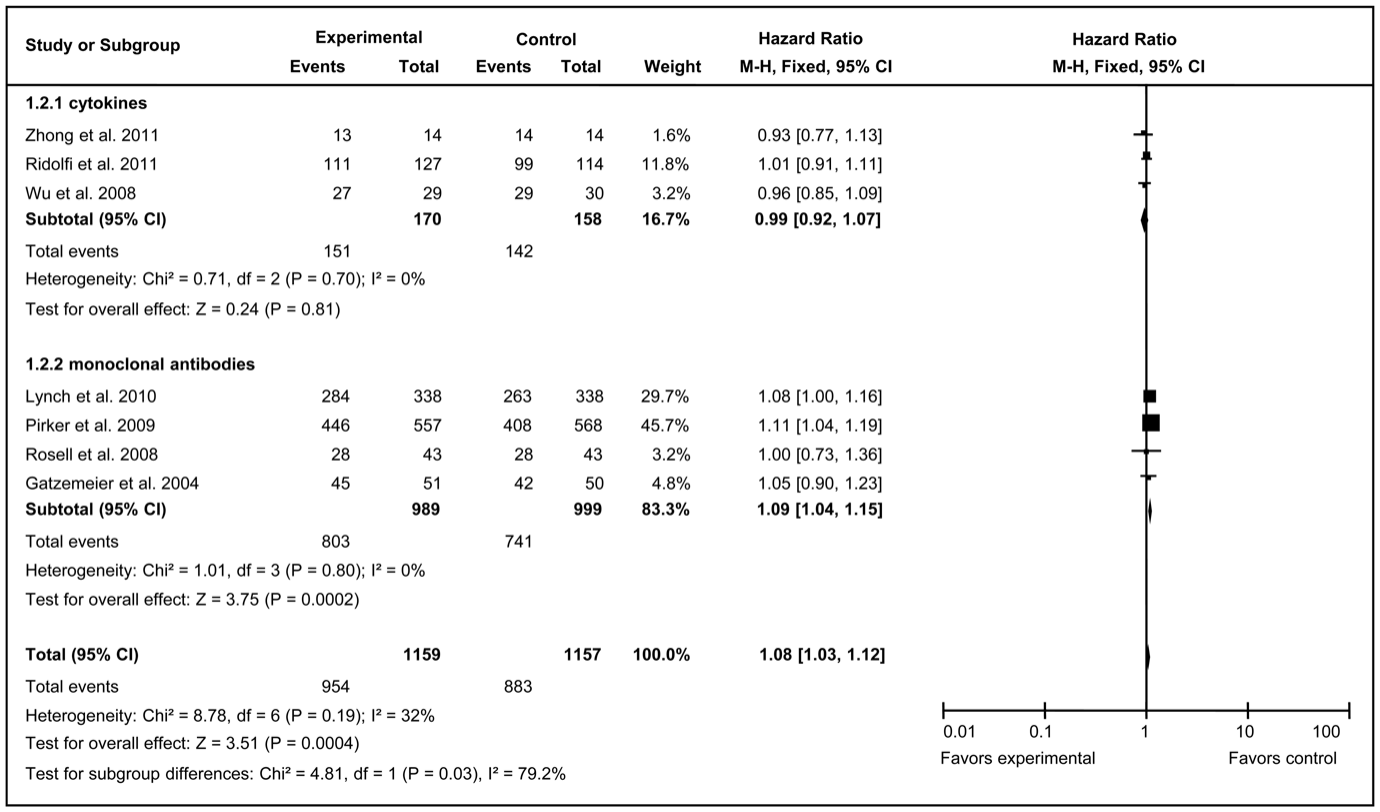
Forest plot of comparison of progression-free survival of 7 included studies (Stage IIIA, IIIB, or IV NSCLC). *P* values are from P-for-effect modification testing for heterogeneity within or across the groups of regimens. The sizes of data markers are proportional to the number of PFS events in the trials. CI, confidence interval; HR, hazard ratio.

Because the vaccine trials did not present PFS data, only the mAb and cytokine groups were subjected to subgroup analysis. No obvious heterogeneity (χ^2^ = 8.78, df = 6, *P* = 0.19; *I* ^2^ = 32%, 95% CI = 0–71%) was detected for total unstratified immunotherapy (Figure 3). Heterogeneity was observed in both the mAb and cytokine groups, allowing the use of different models for the overall and subgroup analyses of PFS. mAbs clearly delayed the time to disease progression (HR = 1.09, 95% CI = 1.04–1.15; *Z = *3.75, *P* = 0.0002), which was consistent with overall immunotherapy (HR = 1.08, 95% CI = 1.03–1.12; *Z* = 3.51, *P* = 0.0004). However, compared with the control group, patients in cytokines group did not have a significant improvement in PFS (HR = 0.99, 95% CI = 0.92–1.07; *Z* = 0.24, *P* = 0.81).

**Figure 4:**
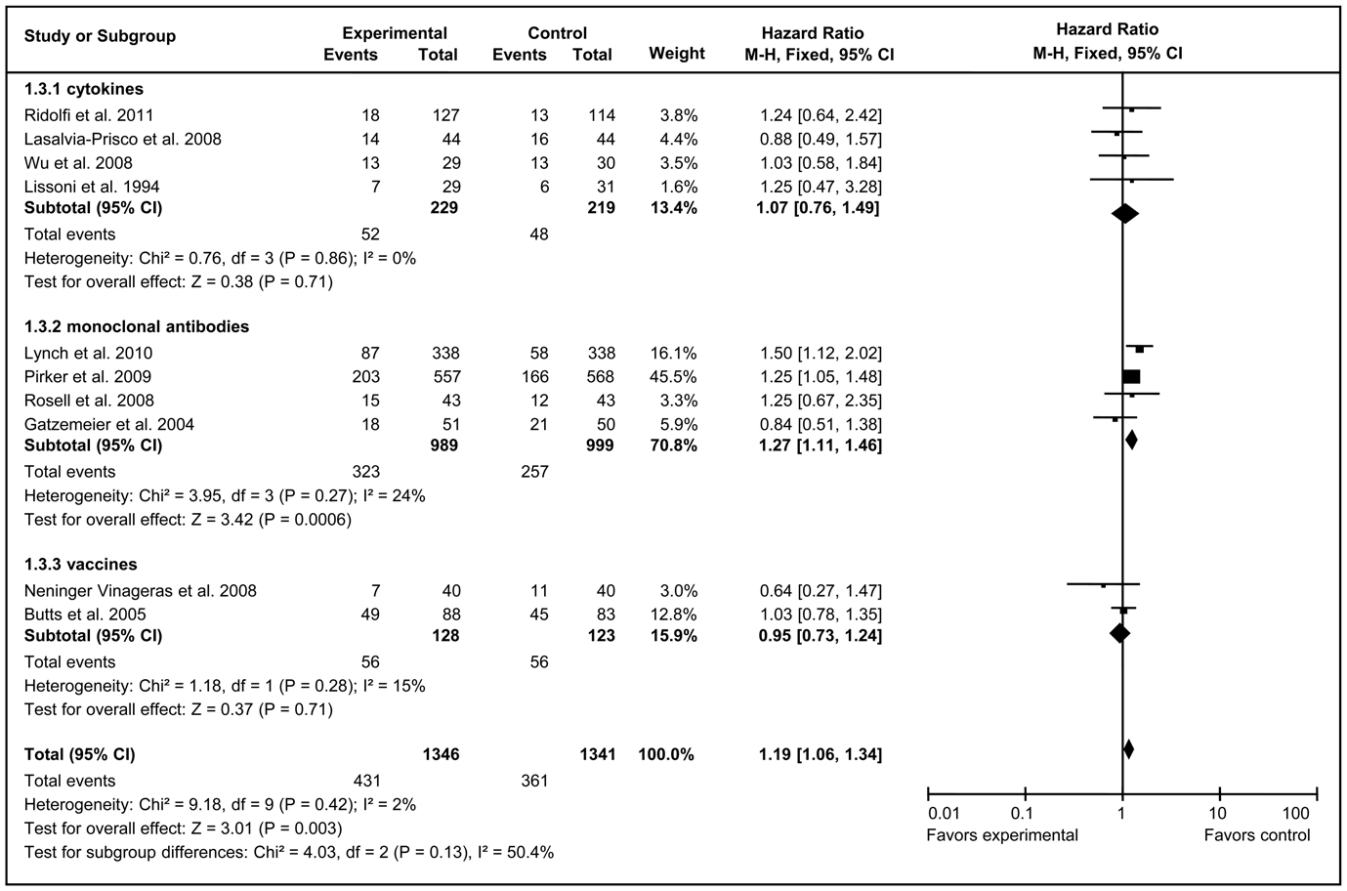
Forest plot of comparison of total effective rate of 10 included studies (Stage IIIA, IIIB, or IV NSCLC). *P* values are from P-for-effect modification testing for heterogeneity within or across the groups of regimens. The sizes of data markers are proportional to the number of total effective rate events in the trials. CI, confidence interval; HR, hazard ratio.

**Figure 5:**
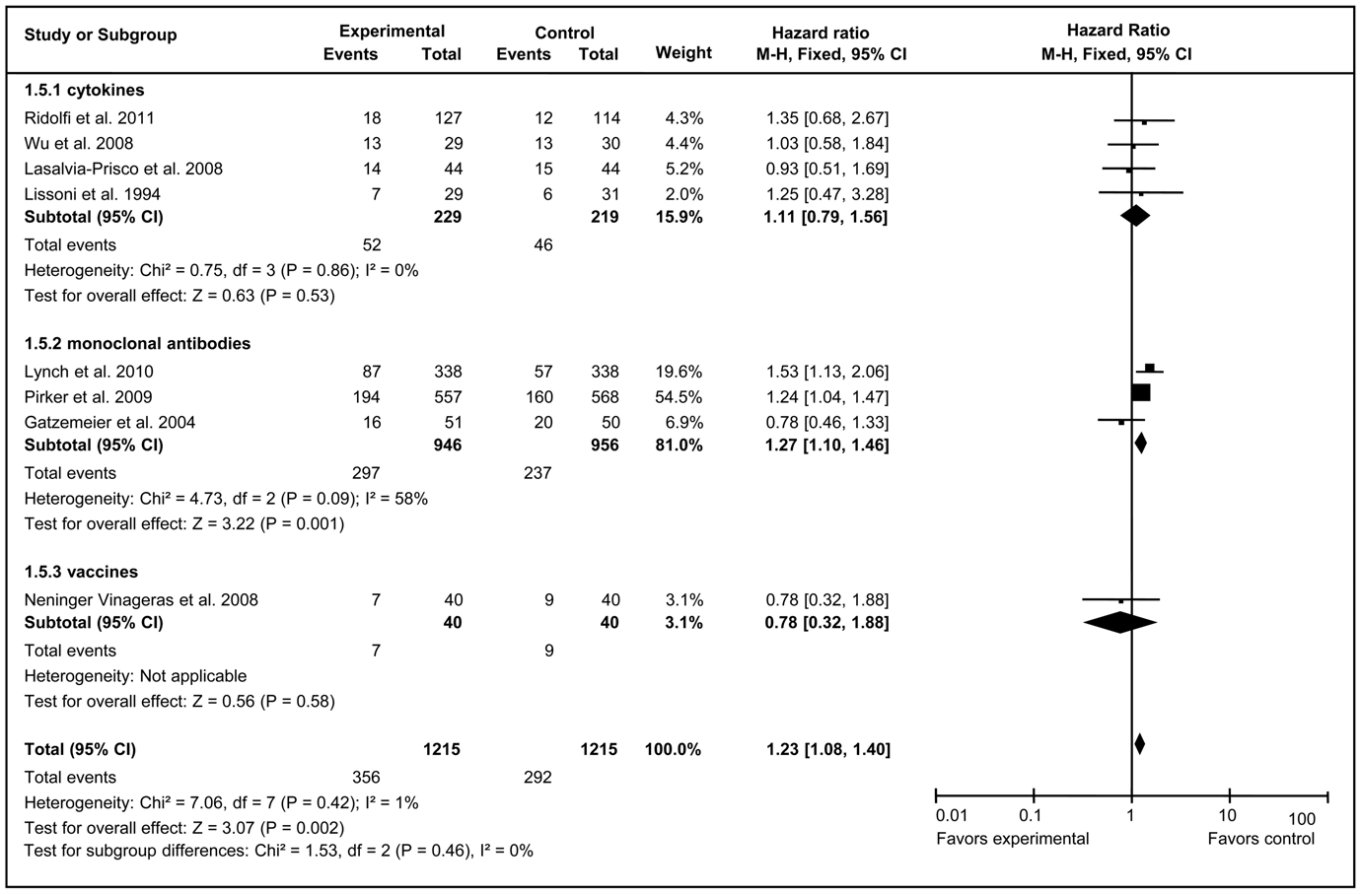
Forest plot of comparison of partial response of 8 included studies (Stage IIIA, IIIB, or IV NSCLC). *P* values are from P-for-effect modification testing for heterogeneity within or across the groups of regimens. The sizes of data markers are proportional to the number of PR rate events in the trials. CI, confidence interval; HR, hazard ratio.

Because of no heterogeneity, fixed-effect models were used to analyze total effective rates and PR rates of total unstratified immunotherapy group and all subgroups (Figures 4 and 5). The overall analysis demonstrated that immunotherapy substantially improved both the total effective rate (HR = 1.19, 95% CI = 1.06–1.34; *Z = *3.01, *P* = 0.003) and PR rate (HR = 1.23, 95% CI = 1.08–1.40, *Z = *3.07, *P* = 0.002) compared with the control arms. mAb therapy significantly improved the total effective rate (HR = 1.27, 95% CI = 1.11–1.46, *Z* = 3.42, *P* = 0.0006) and PR rate (HR = 1.27, 95% CI = 1.10–1.46, *Z* = 3.32, *P* = 0.001), whereas cytokine and vaccine immunotherapy both generated no statistically significant improvement.

**Figure 6:**
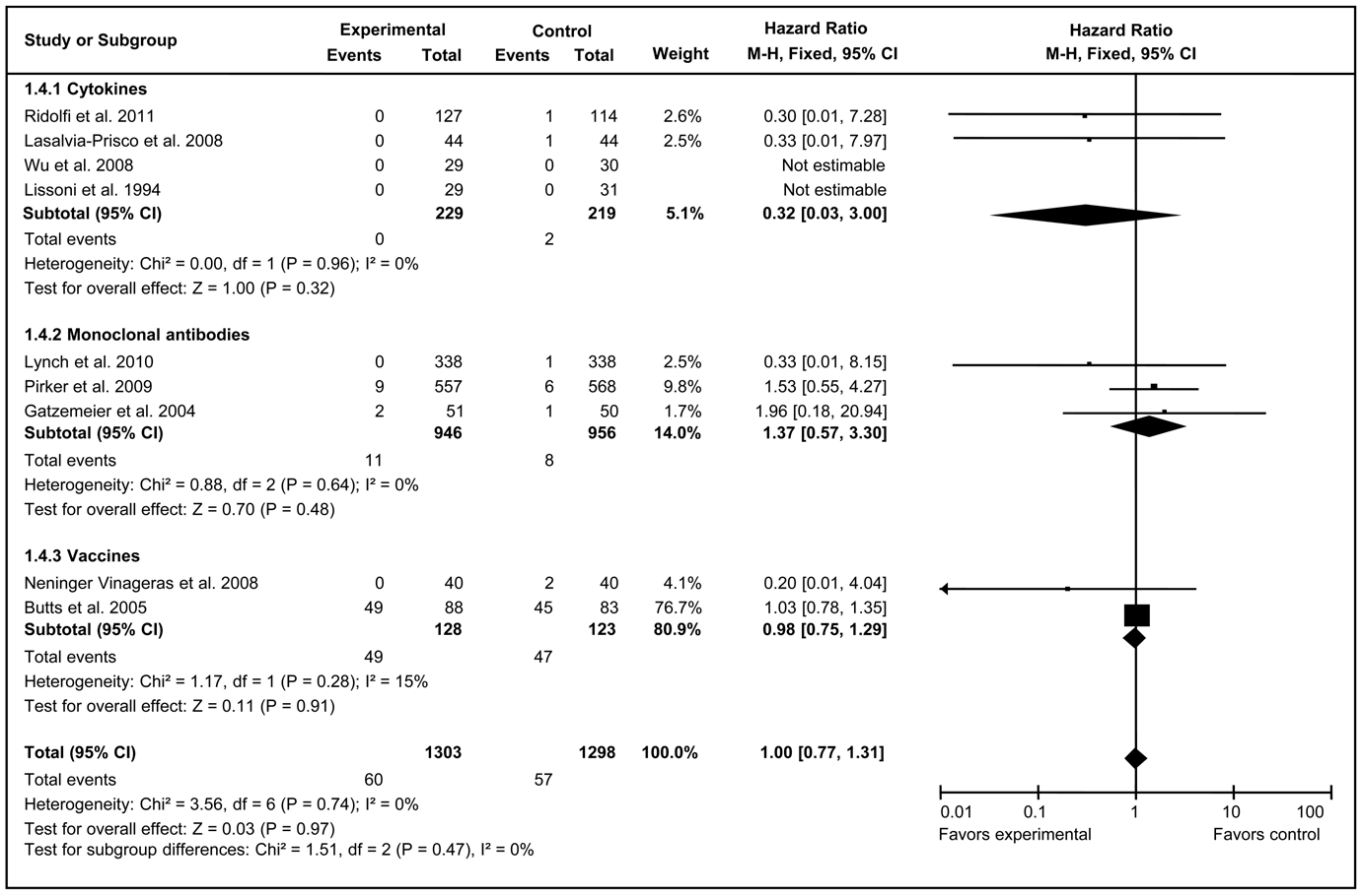
Forest plot of comparison of complete response of 9 included trials (Stage IIIA, IIIB, or IV NSCLC). *P* values are from P-for-effect modification testing for heterogeneity within or across the groups of regimens. The sizes of data markers are proportional to the number of CR rate events in the trials. CI, confidence interval; HR, hazard ratio.

Fixed-effect models were also applied for the analysis of CR in the overall immunotherapy group and three subgroups. The results showed that neither total unstratified immunotherapy (HR = 1.00, 95% CI = 0.77–1.31; *Z = *0.03, *P* = 0.97) nor the immunotherapy subgroups had a significant impact on CR rate compared with their corresponding control arms (Figure 6).

Because not all the efficacy parameters were reported by all the trials reviewed, sensitivity analyses were performed separately on each parameter following the alternative exclusion of the trials by Neninger Vinageras et al. [20] or Butts et al. [21], which did not apply chemotherapy. For the efficacy parameters analyzed, results were all the same to those found in the overall analysis of the pooled trials (Table 3).

**Table 3:** Sensitivity analysis for the outcome of studies not using chemotherapy*.

Excluded trials	Efficacy items	No. of randomized controlled trials subjected to sensitivity analysis [References]	No. of events/Group total subjects		Odds ratio		Heterogeneity test	
			Experimental	Control	Mean (95% CI)	*P*-value	*P*-value	*I* ^2^ (95% CI)
Neninger Vinageras et al.^a^	Overall survival	10 [16], [22], [24]–[31]	1215/1479	1276/1474	0.71 (0.58–0.87)	0.001	0.39	6% (0–65%)
	Total effective rate	9 [16], [22]–[25], [27]–[30]	424/1306	350/1301	1.33 (1.12–1.58)	0.001	0.52	0% (0–65%)
	Complete response	8 [16], [22]–[25], [27], [28], [30]	60/1263	55/1258	1.08 (0.66–1.76)	0.77	0.79	0% (0–75%)
	Partial response	7 [16], [23]–[25], [27], [28], [30]	349/1175	283/1175	1.35 (1.13–1.63)	0.001	0.42	1% (0–71%)
Butts et al.^b^	Overall survival	10 [16], [21], [24]–[31]	1189/1431	1245/1431	0.73 (0.59–0.90)	0.004	0.65	0% (0–62%)
	Total effective rate	9 [16], [21], [23]–[25], [27]–[30]	382/1258	316/1258	1.32 (1.11–1.58)	0.002	0.43	0% (0–65%)
	Complete response	8 [16], [21], [23]–[25], [27], [28], [30]	11/1215	12/1215	0.92 (0.40–2.10)	0.84	0.61	0% (0–75%)

* Statistical heterogeneity was *P*>0.1 for all sensitivity analyses. Sensitivity analysis was conducted according to Peto odds ratio method. CI, confidence interval.

a Neninger Vinageras et al. [21] did not report progression-free survival.

b Butts et al. [22] did not report progression-free survival and partial response.

### Safety

Safety analyses were based on AEs found by the clinical and laboratory examinations in the 12 trials. The treatment-related AEs (grades ≥3) and the immunotherapy efficacy for stage IIIA, IIIB, or IV NSCLC patients are summarized in Tables 4, 5, 6 and 7. Among the 12 trials reviewed, four cytokine and vaccine trials [20], [21], [24], [30] did not provide detailed data or presented somewhat contradictory results on safety. Neninger Vinageras et al. [21] and Wu et al. [30] did not observe serious treatment-related AEs (grade ≥3), whereas Butts et al. [22] and Lissoni et al. [16] reported serious AEs with a significantly less frequently in immunotherapy groups versus control groups. Because various AEs occurred in the other eight trials (Table 4), an overall analysis of safety was conducted. Compared with the control groups, four kinds of serious AEs occurred more frequently in immunotherapy groups: diarrhea, hypomagnesemia, leucopenia, and thrombocytopenia. Six other kinds of AEs occurred equally in the immunotherapy and control groups (Tables 5, 6 and 7). The results indicated that immunotherapy or the combination of immunotherapy with other therapy could lead to different grades of AEs or toxic reactions in patients with advanced NSCLC, and there were fewer episodes of AEs in immunotherapy groups than in non-immunotherapy groups. Diarrhea, hypomagnesemia, and leucopenia occurred more frequently in patients receiving cytokine immunotherapy than in the control group, whereas thrombocytopenia occurred more frequently in the mAb subgroup. Patients receiving vaccine therapy experienced serious AEs with a similar frequency to the control group. With regard to less serious AEs, episodes of non-infectious fever were significantly more frequent in patients receiving immunotherapy than in those receiving chemotherapy in two trials (*P*<0.05, *P* = 0.02, respectively) [16], [31].

**Table 4:** Adverse events (grades ≥3) in advanced NSCLC patients*.

Adverse events	Gatzemeier et al. [23]		Lasalvia-Prisco et al. [24]		Lynch et al. [25]		O'Brien et al. [26]		Pirker et al. [27]		Ridolfi et al. [28]		Rosell et al. [29]		Zhong et al. [31]	
	Exp	Con	Exp	Con	Exp	Con	Exp	Con	Exp	Con	Exp	Con	Exp	Con	Exp	Con
	*n* = 51	*n* = 50	*n* = 44	*n* = 44	*n* = 338	*n* = 338	*n* = 210	*n* = 209	*n* = 557	*n* = 568	*n* = 127	*n* = 114	*n* = 43	*n* = 43	*n* = 14	*n* = 14
Anemia	8	6	ND	ND	17	15	71	54	76	94	ND	ND	6	6	4	6
Leucopenia	17	18	ND	ND	138	97	55	49	139	109	22	19	26	20	10	13
Neutropenia	29	29	5	7	198	177	ND	ND	289	289	58	45	36	23	ND	ND
Thrombocytopenia	18	17	ND	ND	33	29	48	34	ND	ND	64	36	2	1	ND	ND
Nausea	ND	ND	5	7	18	15	ND	ND	ND	ND	22	23	4	3	ND	ND
Hypertension	ND	ND	3	4	ND	ND	ND	ND	ND	ND	ND	ND	ND	ND	ND	ND
Diarrhea	ND	ND	2	1	17	8	ND	ND	25	13	ND	ND	ND	ND	ND	ND
Dyspnea	ND	ND	0	1	ND	ND	ND	ND	47	51	ND	ND	ND	ND	ND	ND
Neurosensory toxicity	ND	ND	5	3	ND	ND	ND	ND	ND	ND	ND	ND	ND	ND	ND	ND
Hypomagnesemia	ND	ND	1	1	26	2	ND	ND	ND	ND	ND	ND	ND	ND	ND	ND

* No treatment-related adverse events (grade ≥3) were observed in trials by Neninger Vinageras et al. [21] and Wu et al. [30]; serious adverse events occurred significantly less frequently in immunotherapy groups than in control groups, but no detailed data were presented in studies by Lissoni et al. [16] and Butts et al. [22].

Note: Exp: experimental group; Con: control group; ND: adverse events (grades ≥3) were not described.

**Table 5:** Adverse events (grade ≥3) in overall immunotherapy and subgroups of advanced NSCLC patients.

*Groups*	*Overall immunotherapy * [*23*]–[29], [31]			*Cytokines subgroup * [*23*], [25], [27], [29]			*Vaccines subgroup * [*26*]			*mAbs subgroup * [*24*], [28], [31**]		
Adverse events	OR	95% CI	*P*-value	OR	95% CI	*P*-value	OR	95% CI	*P*-value	OR	95% CI	*P*-value
Anemia	1.00	0.80–1.25	0.9787	0.88	0.67–1.16	0.3659	1.47	0.96–2.23	0.0752	0.61	0.17–2.20	0.4522
Diarrhea	2.03	1.21–3.40	**0.0074**	2.07	1.21–3.51	**0.0075**	/	/	/	1.87	0.17–20.80	0.6110
Dyspnea	0.90	0.60–1.34	0.5987	0.93	0.62–1.39	0.72	/	/	/	0.31	0.01–7.62	0.4720
Hypertension	0.75	0.17–3.34	0.7032	/	/	/	/	/	/	0.69	0.15–3.14	0.6335
Hypomagnesemia	9.13	2.76–30.17	**0.0003**	13.46	3.19–56.86	**0.0004**	/	/	/	0.93	0.06–14.97	0.9588
Leucopenia	1.35	1.14–1.60	**0.0005**	1.48	1.22–1.80	**0.0001**	1.16	0.74–1.81	0.5155	0.92	0.53–1.57	0.7477
Nausea	1.02	0.68–1.53	0.9292	1.24	0.66–2.33	0.5030	/	/	/	0.81	0.46–1.43	0.4635
Neurosensory toxicity	1.66	0.40–6.98	0.4861	/	/	/	/	/	/	1.56	0.37–6.65	0.5441
Neutropenia	1.14	0.98–1.32	0.0962	1.17	0.98–1.40	0.0765	/	/	/	1.19	0.76–1.86	0.4403
Thrombocytopenia	1.46	1.14–1.88	**0.0029**	1.15	0.77–1.72	0.5049	1.53	0.94–2.49	0.0904	2.00	1.24–3.22	**0.0044**

Note: mAbs, monoclonal antibodies; OR, odds ratio. CI, confidence interval. No treatment-related adverse events (grade ≥3) were observed in trials by Neninger Vinageras et al. [21] and Wu et al. [30]; serious adverse events occurred but significantly less frequently in immunotherapy groups than in control groups and no detailed data were presented in studies by Lissoni et al. [16] and Butts et al. [22].

**Table 6:** Adverse events (grade ≥3) in overall immunotherapy and subgroups of advanced NSCLC patients.

*Overall immunotherapy * [*23]*–[29], [31**]	*Experimental (N = 1384)*	*Control (N = 1380)*	*Odds ratio*	*95% CI*	*P-value*
Anemia	182	181	1.0030	0.80–1.25	0.9787
Diarrhea	44	22	2.0269	1.21–3.40	**0.0074**
Dyspnea	47	52	0.8978	0.60–1.34	0.5987
Hypertension	3	4	0.7473	0.17–3.34	0.7032
Hypomagnesemia	27	3	9.1326	2.76–30.17	**0.0003**
Leucopenia	407	325	1.3523	1.14–1.60	**0.0005**
Nausea	49	48	1.0185	0.68–1.53	0.9292
Neurosensory toxicity	5	3	1.6642	0.40–6.98	0.4861
Neutropenia	615	570	1.1365	0.98–1.32	0.0962
Thrombocytopenia	165	117	1.4612	1.14–1.88	**0.0029**

Adverse event items in cytokines subgroup [23], [25], [27], [29]	Experimental (*N* = 989)	Control (*N* = 999)	Odds ratio	95% CI	*P*-value
Anemia	107	121	0.88	0.67–1.16	0.3659
Diarrhea	42	21	2.07	1.21–3.51	**0.0075**
Dyspnea	47	51	0.93	0.62–1.39	0.72
Hypertension	ND	ND	/	/	/
Hypomagnesemia	26	2	13.46	3.19–56.86	**0.0004**
Leucopenia	320	244	1.48	1.22–1.80	**0.0001**
Nausea	22	18	1.24	0.66–2.33	0.5030
Neurosensory toxicity	ND	ND	/	/	/
Neutropenia	552	518	1.17	0.98–1.40	0.0765
Thrombocytopenia	53	47	1.15	0.77–1.72	0.5049

Adverse event items in vaccines subgroup [26]	Experimental (*N* = 210)	Control (*N* = 209)	Odds ratio	95% CI	P-value
Anemia	71	54	1.47	0.96–2.23	0.0752
Diarrhea	ND	ND	/	/	/
Dyspnea	ND	ND	/	/	/
Hypertension	ND	ND	/	/	/
Hypomagnesemia	ND	ND	/	/	/
Leucopenia	55	49	1.16	0.74–1.81	0.5155
Nausea	ND	ND	/	/	/
Neurosensory toxicity	ND	ND	/	/	/
Neutropenia	ND	ND	/	/	/
Thrombocytopenia	48	34	1.53	0.94–2.49	0.0904

Adverse event items in mAbs subgroup [24], [28], [31]	Experimental (*N* = 185)	Control (*N* = 172)	Odds ratio	95% CI	*P*-value
Anemia	4	6	0.61	0.17–2.20	0.4522
Diarrhea	2	1	1.87	0.17–20.80	0.6110
Dyspnea	0	1	0.31	0.01–7.62	0.4720
Hypertension	3	4	0.69	0.15–3.14	0.6335
Hypomagnesemia	1	1	0.93	0.06–14.97	0.9588
Leucopenia	32	32	0.92	0.53–1.57	0.7477
Nausea	27	30	0.81	0.46–1.43	0.4635
Neurosensory toxicity	5	3	1.56	0.37–6.65	0.5441
Neutropenia	63	52	1.19	0.76–1.86	0.4403
Thrombocytopenia	64	36	2.00	1.24–3.22	**0.0044**

Note: No treatment-related adverse events (grade ≥3) were observed in trials by Neninger Vinageras et al. [21] and Wu et al. [30]; serious adverse events occurred but significantly less frequently in immunotherapy groups versus control groups and no details were provided by Lissoni et al. [16] and Butts et al. [22]. mAbs, monoclonal antibodies; CI, confidence interval. ND: the corresponding adverse events (grades ≥3) were not described.

**Table 7:** Efficacy analysis on overall immunotherapy and subgroups.

Efficacy parameters	Overall immunotherapy	Vaccine	Cytokine	Monoclonal antibody
Overall survival	SS	SS	NS	S
Progression-free survival^a^	SS	ND	NS	SS
Total effective rate	SS	NS	NS	SS
Partial response	SS	NS	NS	SS
Complete response	NS	NS	NS	NS
Adverse events^b^	++++	NS/ND	+++	+

S, barely significant with *P* = 0.05.

SS, substantially significant difference at 0.01 level between immunotherapy arm and the corresponding control arm.

NS, no significant difference compared to the corresponding control arm.

ND, no detailed data were described for.

a progress-free survival in vaccine-adopted trials and.

b adverse events (grades ≥3) in four vaccine or cytokine trials [16], [21], [22], [30].

Each plus (+) represents one kind of adverse event (grades ≥3) for which the immunotherapy arm was significantly greater.

## Discussion

The 12 trials included in this meta-analysis adopted three kinds of immunotherapy (vaccines, cytokines, mAbs) for advanced NSCLC patients. Hence the number of published randomized controlled trials for each kind of immunotherapy would affect the results of this study. The quality of the reported data influenced the power of our meta-analysis, and greater statistical reliability would be achieved if additional and more comprehensive trials including all of the efficacy parameters were enrolled. Nevertheless, sensitivity analyses on the various efficacy parameters with alternative exclusions of one of the trials supported the conclusions drawn from the overall unstratified analyses. Other factors, such as race differences of patients, curative agents administrated simultaneously with immunotherapy, different immunotherapy strategies, different lengths of follow-up, and different proportions lost to follow-up may confer limitations on this meta-analysis. In overall studies, no significant publication bias existed [32]. To avoid bias in the identification and selection of studies, as many randomized controlled trials as possible were included to improve the statistical reliability. Our literature search strategy guaranteed that there was less possibility of important published trials being overlooked. According to our meta-analysis, all patients with advanced NSCLC met quality-control specifications and protocol eligibility [16], [21]–[31]. Subgroup analyses were conducted according to recently proposed criteria [33], [34], and their validity was enhanced by the fine discrimination of the subgroups of 12 immunotherapy trials. Finally, Kaplan-Meier estimation of hazard ratios demonstrated that no statistical inconsistency existed between the results from each of the original studies and those of the overall or subgroup analyses of immunotherapy efficacy, suggesting that the results of this meta-analysis are valid.

Roughly two-thirds of lung cancer patients have locally advanced or disseminated diseases, and surgery is not adopted at the time of diagnosis [3]. Therefore, efficient alternative therapy is needed. The results of the overall meta-analysis showed that immunotherapy significantly improved the PFS, total effective rate, and PR rate (*P* = 0.0004, 0.003, 0.002, respectively) in despite of less influence on the CR rate (*P* = 0.97), suggesting that immunotherapy may provide advantages for patients with advanced NSCLC. However, immunotherapeutic approaches in the treatment of NSCLC were always applied based on standard treatment modalities or in combination with multiple immunotherapeutic agents rather than as single-agent therapy [16], [21]–[31]. Subgroup analyses showed that only mAb-treated group significantly benefited from immunotherapy with regard to PFS, total effective rate, and PR rate (*P* = 0.0002, 0.0006, 0.001, respectively), with a trend of improvement in OS (*P* = 0.05). The vaccine-treated group achieved significant improvement only in OS (*P* = 0.009), and cytokines-treated group did not significantly improve OS, total effective rate, PR rate (*P* = 0.08, 0.81, 0.71, respectively). Furthermore, all three subgroups did not improve the CR rates (Table 6).

Vaccine and cytokine immunotherapies are novel modalities for the treatment of advanced NSCLC [21], [26], [31], [35]–[38], and specific immune responses have been documented in many advanced NSCLC studies [9], [37]. Our meta-analysis showed, however, that no significant clinical efficacy was achieved by the two kinds of immunotherapy when they were applied to advanced NSCLC patients. In addition, cytokine immunotherapy significantly induced several kinds of treatment-related AEs. Taken together, mAb immunotherapy was considered to be the most potential therapy for advanced NSCLC patients compared with other immunotherapy strategies.

The importance of AEs and toxicity must be emphasized. Although mAb immunotherapy could improve efficacy, more AEs or toxicity occurred in mAb immunotherapy groups than in control groups, which may discount the efficacy of immunotherapy and lower the CR rate. To further improve the efficacy of immunotherapy, researchers should develop immunotherapeutic regimens to reduce or eliminate toxicity and AEs, which can further improve the quality of life of advanced NSCLC patients. In fact, aside from individual differences, drug dose, and administration protocols, the molecular structure of the drug protein [39] is the most important factor related to efficacy and safety of immunotherapy.

In the past two decades, 32 mAb drugs have been approved by U.S. Food and Drug Administration, but two of the three drugs that could be involved in preclinical trials have been withdrawn from the market due to their serious adverse events in human patients [40]. The mAbs can be quickly developed and demonstrated to be efficacious for advanced NSCLC patients. However, mAbs immunotherapy-associated AEs and anaphylaxis could timely occur [23], [25], [27], [29] or be delayed with the treatment process [41]. To minimize AEs or anaphylaxis, further researches on two aspects are merited. First, because even mAb containing less than 10% mouse-derived fragments (i.e., ≥90% humanized) can result in AEs [42], fully humanized mAbs should be developed to eliminate mouse epitopes. Second, the allergenicity of mAbs should be further attenuated and/or eliminated. The resolution of these two issues would allow the development of more efficacious and safer agents for immunotherapy treatment of advanced NSCLC.

In conclusion, anticancer therapy should be performed based on an individual assessment of the risk of recurrence and death caused by the therapy, i.e. the balance between toxicity and efficacy, and even changes in quality of life [43]. The efficacy and safety of new therapies must be assessed appropriately for physicians to decide how to select the optimal treatment strategy. We found that immunotherapy using mAbs, rather than cytokines and vaccines, could significantly improved PFS, total effective rate, and PR rate, suggesting that mAb immunotherapy may become a standard complementary therapeutic approach for advanced NSCLC patients in the future. In despite of this, more efficacious and safer (i.e., causing fewer AEs and less allergenicity) immunotherapeutic agents should also be developed.
